# Declining Orangutan Encounter Rates from Wallace to the Present Suggest the Species Was Once More Abundant

**DOI:** 10.1371/journal.pone.0012042

**Published:** 2010-08-11

**Authors:** Erik Meijaard, Alan Welsh, Marc Ancrenaz, Serge Wich, Vincent Nijman, Andrew J. Marshall

**Affiliations:** 1 People and Nature Consulting International, Jakarta, Indonesia; 2 School of Archaeology and Anthropology, Australian National University, Canberra, Australian Capital Territory, Australia; 3 Centre for Mathematics and its Applications, Mathematical Sciences Institute, Australian National University, Canberra, Australian Capital Territory, Australia; 4 Kinabatangan Orangutan Conservation Project, Sandakan, Sabah, Malaysia; 5 Anthropological Institute & Museum, University of Zurich, Zurich, Switzerland; 6 Sumatran Orangutan Conservation Programme (PanEco-YEL), Medan, Sumatra, Indonesia; 7 Zoological Museum Amsterdam, Amsterdam, The Netherlands; 8 Department of Anthropology and Geography, School of Social Sciences and Law, Oxford Brookes University, Headington, Oxford, United Kingdom; 9 Department of Anthropology, Graduate Group in Ecology and Animal Behavior Graduate Group, University of California Davis, Davis, California, United States of America; Stanford University, United States of America

## Abstract

**Background:**

Bornean orangutans (*Pongo pygmaeus*) currently occur at low densities and seeing a wild one is a rare event. Compared to present low encounter rates of orangutans, it is striking how many orangutan each day historic collectors like Alfred Russel Wallace were able to shoot continuously over weeks or even months. Does that indicate that some 150 years ago encounter rates with orangutans, or their densities, were higher than now?

**Methodology/Principal Findings:**

We test this hypothesis by quantifying encounter rates obtained from hunting accounts, museum collections, and recent field studies, and analysing whether there is a declining trend over time. Logistic regression analyses of our data support such a decline on Borneo between the mid-19th century and the present. Even when controlled for variation in the size of survey and hunting teams and the durations of expeditions, mean daily encounter rates appear to have declined about 6-fold in areas with little or no forest disturbance.

**Conclusions/Significance:**

This finding has potential consequences for our understanding of orangutans, because it suggests that Bornean orangutans once occurred at higher densities. We explore potential explanations—habitat loss and degradation, hunting, and disease—and conclude that hunting fits the observed patterns best. This suggests that hunting has been underestimated as a key causal factor of orangutan density and distribution, and that species population declines have been more severe than previously estimated based on habitat loss only. Our findings may require us to rethink the biology of orangutans, with much of our ecological understanding possibly being based on field studies of animals living at lower densities than they did historically. Our approach of quantifying species encounter rates from historic data demonstrates that this method can yield valuable information about the ecology and population density of species in the past, providing new insight into species' conservation needs.

## Introduction

Historical knowledge of species is vital to prevent what is known as the ‘shifting baseline syndrome’ [1]. This occurs because most species and ecosystems are assessed by scientists only after long periods of exploitation. The resulting historic amnesia leads us to consider current degraded systems or reduced species densities as representative of the recent evolutionary past. The syndrome has been assessed for some marine systems in western countries, for which the historical record is relatively rich [2]. For species in tropical forest systems, to our knowledge no such analyses exist. We present a new approach to assess the shifting baseline for a species of high conservation concern, the Bornean orangutan (*Pongo pygmaeus*).

Orangutans live at population densities that rarely exceed 5 animals/km^2^ and are typically below 2.5 animals/km^2^ [3]. In their natural forest habitat in Borneo and Sumatra, unhabituated animals can be difficult to find because of their generally dispersed and cryptic nature. Field scientists mostly encounter them alone or in groups of 2 or 3 individuals, while larger groups are seen only rarely in times of high orangutan food availability. The low population densities, as well as the related low sociality currently observed in wild orangutans, are generally thought to have characterised their evolutionary history, but this remains untested. Accounts by nineteenth century explorers indicate that wild orangutans may have lived at substantially higher densities in the recent past than they do now. For example, the famous naturalist Alfred Russel Wallace [4] quite easily collected 29 orangutans during his stay in Malaysian Borneo in 1855. Beccari [5] shot or saw 26 individuals in a period of 37 days in the forest. Selenka [6] did not keep clear records of his collection activities, but the approximately 400 orangutan specimens that he collected between 1892 and 1895 testify the relative ease with which he found them. In 1912, explorers reportedly saw 35 wild orangutans in one day along the Kinabatangan River in Sabah, Malaysia [7].

Previous orangutan fieldworkers have discussed the possibility that recent orangutan encounter rates are substantially lower than those reported in historical records [8], [9], [10], even in forests that had not been disturbed by timber harvest or fire. Schaller [10] noted that Hornaday [11] not infrequently encountered animals twice in the same day while travelling along rivers in Sarawak, Malaysian Borneo, on which Schaller saw only scattered nests during his study.

These anecdotal observations suggest that historic orangutan encounter rates could have been substantially higher than recent ones. If that is correct, it could indicate that, in the past, orangutans lived at higher population densities than now. The large body of literature on orangutan ecology and behaviour, however, carries the tacit assumption that present-day orangutan densities in little or undisturbed forest are at their ecological carrying capacity, determined by habitat-specific resource availability [12], [13], [14], [15], [16]. Evidence of a recent historical decline in orangutan densities would challenge this premise, with important implications for conservation and our understanding of orangutan socio-ecology.

Here we explicitly test the hypothesis that orangutan population densities in areas with little or no anthropogenic habitat disturbance have declined significantly over the last 150 years. We test the hypothesis by assessing changes in daily orangutan encounter rates over the last 150 years. We use the historic literature and museum records from orangutan hunting and survey expeditions in Borneo to estimate orangutan encounter rates, and apply robust statistical approaches to evaluate whether these rates have decreased over time. Such approaches remain rare [e.g., [17], [18]] and are largely untested in their usefulness to conservation. If effective they could provide an important new tool in species conservation management.

## Results

**Figure 1:**
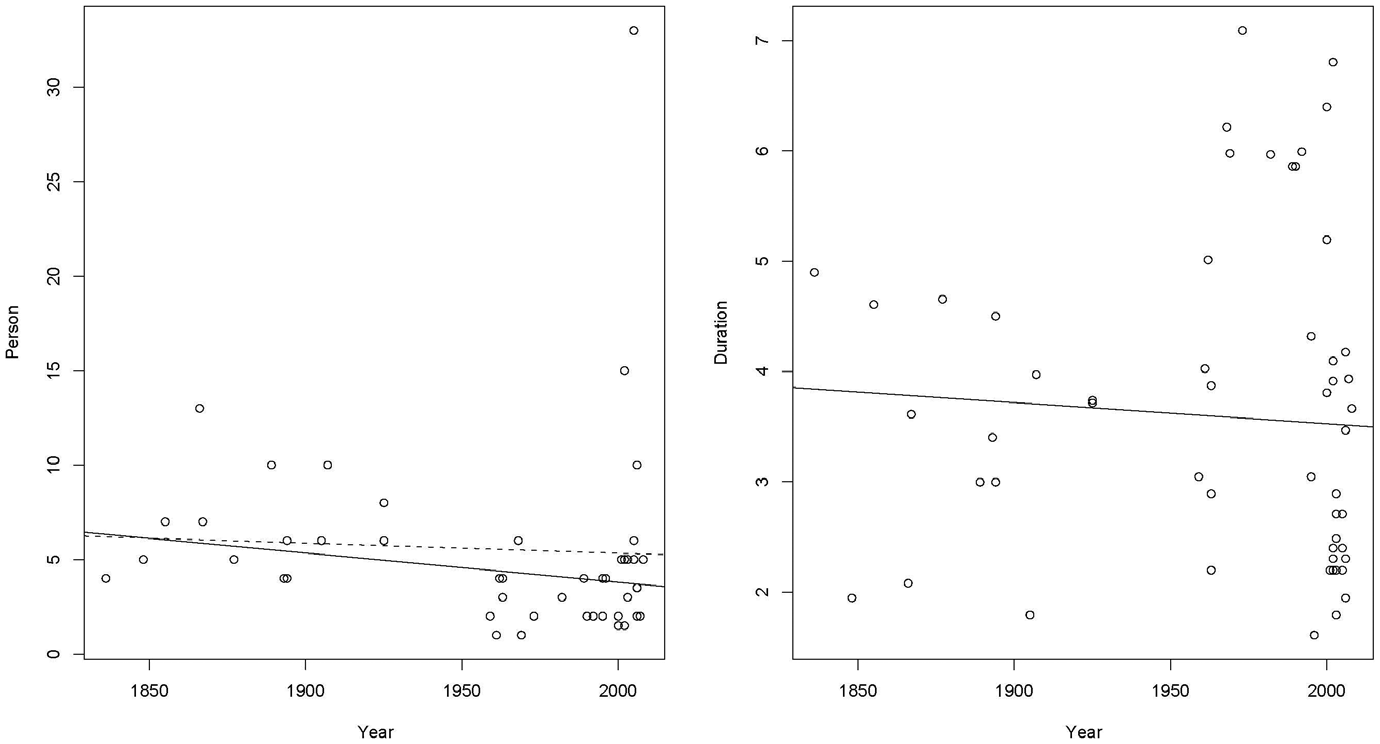
Expedition size and duration over time. The relationship between expedition size (measured in the number of people) and year and between expedition duration (measured in log(*Days*)) and *Year*. Figure 1a shows the decreasing trend in expedition size over time. The dashed line is the least squares regression line and the solid line is the fit from a robust procedure which excludes the outlying 33 person expedition. The effect of excluding the 33 person expedition is to increase the rate of decrease in expedition size. Figure 1b shows the decreasing trend in expedition duration over time.

We gathered data on 77 Bornean expeditions and surveys (Supporting Information, Table S1), of which 59 contain details about the expedition size; our full analysis is based on these 59 expeditions. Orangutans were detected on 43 of these expeditions. We first explored whether expedition size has changed over time (Fig. 1a). Overall, expedition size has tended to decrease across time, with a notable exception the expedition of 2005 with 33 people, which is exceptionally large and stands out as an outlier. We fitted a linear regression relating the size to time. The decrease in size is small and not significant, but a robust fit using Tukey's bisquare estimator [e.g., [19], p. [51]] which downweights the 33 person expedition shows a significant decline in expedition size with time. This shows that it is potentially important in the analysis to adjust for the expedition size. Similarly, we looked at whether expedition duration has changed over time (Fig. 1b). There are a number of long trips after 1950, but there are also many more short ones and the overall trend is for trip duration to decrease with time. As with expedition size, there was a need to adjust for trip duration in the analysis.

We assessed the probability of detecting at least one orangutan on an expedition by fitting logistic regression models. We used a binary regression model (-detecting or not detecting an orangutan) in which p_i ( = Probability of detecting at least one orangutan on the i^th^ expedition) was modelled as:
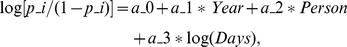
with *Year* = year of the expedition, *Person* = expedition size, and *Days* = duration of the expedition.

The results of fitting this model are shown in Table 1 (Model 1a). Because the 33-person expedition is an outlier (i.e., much larger than the other expeditions, with the next largest 15 people), we decided to exclude it from the analysis, which results in fairly similar regression coefficients (Model 1b). The number of persons on an expedition and to a lesser extent the year of an expedition are more important in this second model (Table 1, Model 1b). The model shows that the probability of detecting at least one orangutan decreases with year and expedition size, but increases with the duration of the expedition. Only the duration of the expedition is significant.

**Table 1:** Logistic regression model for probability of detecting at least one orang-utan on an expedition.

	Estimate	Std. Error	z value	Pr(>|z|)
Intercept Model 1a	18.41009	16.85967	1.092	0.27485
Intercept Model 1b	26.636752	17.933663	1.485	0.13747
Year Model 1a	−0.01174	0.00852	−1.378	0.16820
Year Model 1b	−0.015438	0.009071	−1.702	0.08879
Person Model 1a	0.02789	0.06676	0.418	0.67605
Person Model 1b	−0.164621	0.125249	−1.314	0.18873
log(Days) Model 1a	1.92088	0.63607	3.020	0.00253 **
log(Days) Model 1b	1.965192	0.639650	3.072	0.00212 **

Model 1a includes the 33 person expedition. Model 1b excludes 33 person expedition. *Year* = year in which expedition was conducted. *Person* = number of people on an expedition. Log(*Days*) = natural logarithm of duration of expedition in days. Significance code: ‘**’: p<0.01.

To assess the influence of the number of people on the expedition and year of the expedition, we refitted the model first omitting Person and then Year (Supporting Information, Table S2). There is not much change in the coefficient of duration or its standard error. The conclusion is that the most important factor affecting the probability of detecting at least one orangutan is the duration of the expedition (Supporting Information, Fig. S1). There is weak evidence that after adjusting for duration, the probability of detecting at least one orangutan is decreasing over time.

**Figure 2:**
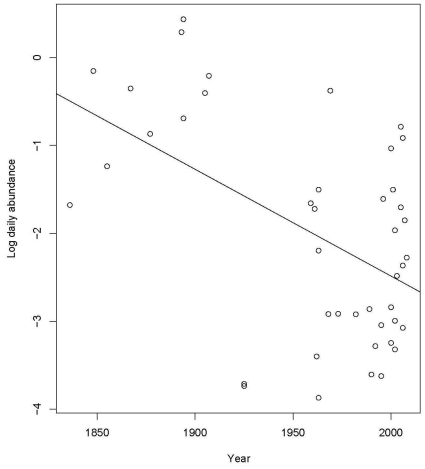
Changes in daily abundance over time. The relationship between daily abundance and year conditional on at least one encounter during the expedition, showing the decreasing trend in log daily abundance over time.

It is possible that reports of expeditions which did not encounter orangutans did not specifically mention this. We therefore explored the relationship of abundance to the same variables (*Year*, *Person*, *Days*), conditional on detection. To be consistent with the detection analysis, we used the actual (non-zero) number of orangutans seen or shot during an expedition and fitted the linear regression model:
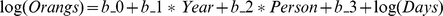
This model fits quite well except that there is some evidence of increasing variability in the residuals. This is apparent in the residual plot but less so in the scale-location plot (Supporting Information, Fig. S2). Fitting a linear model:
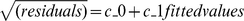
gives an estimate of c_1 of 0.02 which is very small and suggests the heteroscedasticity is not severe. We did try to remove even this small effect, but other transformations of orangutan encounter rates produce worse fit and simple weighting seems to have no effect on the diagnostics while making the model much more complicated. Conditional on at least one detection, the abundance of orangutans decreases with year but increases with expedition size and duration (Table 2, Model 2a, Fig. 2). Both year and duration are significant. Omitting expedition size has essentially no effect on the model (Table 2).

**Table 2:** Linear regression model for orang-utan abundance on an expedition, conditional on at least one detection.

	Estimate	Std. Error	z value	Pr(>|z|)
Intercept Model 2a	21.500714	5.689382	3.779	0.000527 ***
Intercept Model 2b	21.487783	5.685365	3.779	0.000513 ***
Year Model 2a	−0.011465	0.002917	−3.931	0.000337 ***
Year Model 2b	−0.011317	0.002911	−3.888	0.000372 ***
Person Model 2a	0.028371	0.029204	0.971	0.337299
log(Days) Model 2a	0.708796	0.108887	6.509	1.02e-07 ***
log(Days) Model 2b	0.677241	0.103856	6.521	8.76e-08 ***

Model 2a provides full model of non-zero abundance. Model 2b provides model of non-zero abundance, omitting Person. *Year* = year in which expedition was conducted. *Person* = number of people on an expedition. Log(*Days*) = natural logarithm of duration of expedition in days. Residual standard error in Model 2a: 0.97 on 39 degrees of freedom. Residual standard error in Model 2b: 0.9693 on 40 degrees of freedom. Significance code: ‘***’: p<0.001.

A simpler model is obtained by setting the coefficient of log(*Day*s) equal to 1 and fitting a linear model to log(*Orangs*1/*Days*1), the logarithm of the daily abundance. This approach improves the fitting diagnostics (Table 3, Model 3a), and gives results which are consistent with the above analysis: the trend is decreasing significantly with year and increasing with expedition size although this latter effect is not significant. Again, leaving out the non-significant variable has negligible effect (Table 3, Model 3b). This strong agreement and supporting diagnostic plots for the model (Supporting Information, Fig. S3) reinforce the conclusion from the first analysis: Conditional on at least one detection, the abundance of orangutans decreases with year but increases with expedition duration.

**Table 3:** Linear regression model for orang-utan abundance on an expedition, conditional on at least one detection setting the coefficient of log(Days) equal to 1.

	Estimate	Std. Error	z value	Pr(>|z|)
Intercept Model 3a	22.052884	6.151321	3.585	0.000906 ***
Intercept Model 3b	21.80096	6.25595	3.485	0.001187 **
Year1 Model 3a	−0.012431	0.003137	−3.962	0.000298 ***
Year1 Model 3b	−0.01214	0.00319	−3.807	0.000462 ***
Person1 Model 3a	0.048302	0.030142	1.602	0.116922

Model 3a provides full model of non-zero abundance. Model 3b provides model of non-zero abundance, omitting Person. *Year* = year in which expedition was conducted. *Person* = number of people on an expedition. Log(*Days*) = natural logarithm of duration of expedition in days. Residual standard error in Model 3a: 1.049 on 40 degrees of freedom. Residual standard error in Model 3b: 1.067 on 41 degrees of freedom. Significance codes: ‘**’: p<0.01; ‘***’: p<0.001.

To calculate the decline in abundance we use model 3b, so that we estimate the expected log daily abundance. The estimates with 95% confidence intervals are: 1850 (log(*Orangutans*/day = −0.0664132; [−1.447832–0.1195680]) and 2005 (log(*Orangutans*/day = −2.546343; [−2.982631–−2.1100545]). These are confidence intervals rather than prediction intervals which would be appropriate for predicting an observation rather than the expected daily response. We can back transform these to the raw scale to get: 1850 (*Orangutans*/day = 0.51; [0.23–1.13]) and 2005 (*Orangutans*/day = 0.08; [0.05–0.12]). In other words, in 1850, one could encounter one orangutan on average every second day, whereas 155 years later, this had declined to one orangutan every 13 days.

## Discussion

### Encounter rates and densities

It is suggested herein there has been a decline in orangutan encounter rates on Borneo between the mid 19^th^ century and the present, with mean daily encounter rates appearing to have declined about 6-fold in areas with little or no forest disturbance. We do not know whether this indicates a decrease in orangutan densities of the same magnitude, because encounter rates and densities may not be linearly related. For example, if orangutans that occur at higher densities tended to exhibit a more clumped distribution (e.g., due to aggregation around high quality food resources), then encounter rates may increase exponentially at high population densities. There is some support of decreasing maximum group size, with two historic hunters reporting encountering groups of 7 animals [5], [20]. Such group sizes have not been reported on Borneo in recent times, although 4 respondents reported seeing 5 animals together. Without further information on the relationship between encounter rates and densities it is not possible to estimate historic densities of orangutans.

### Possible sources of bias

We recognize that the data have limitations, and the various biases that are introduced by comparing historic literature and museums records to recent field surveys are caveats to any of our conclusions. Some possible sources of bias such as the different durations and different sizes of expeditions are easily identified and, when these variables are known, can be included as parameters in our models. Other sources of bias are less easy to remove.

One obvious question is whether historic hunting and surveying methods are similar enough to present-day surveys to warrant comparison. Museum collectors used local trackers to find and shoot orangutans and other species, or to notify the collectors of the presence of orangutans so that the collectors could shoot them. Surveyors, both recent and historic, would walk through a forest area and note the orangutans they encountered. The focus in present-day transect surveys is often on orangutan nests rather than the animal itself, and this method may reduce the chance of orangutan encounters. To test for this effect, we subsampled our data and only selected those recent surveys in which the specific purpose was to sample quietly a forest area with a team of surveyors and count directly as many orangutans as possible. Nine surveys in different parts of Borneo between 2002 and 2009, involving 724 surveys days, resulted in the detection of 108 orangutans, or a daily encounter rate of 0.18 (SD = 0.22). This is about double the estimated average encounter rate for recent surveys based on the full data set (0.08), suggesting that normal nest transect surveys reduce the likelihood of encountering orangutans. Still, this estimate is three times lower than the historic encounter rates, even though the detection methods are similar.

Under-reporting of non-encounters with orangutans is another possible source of bias. Hunters mostly focused their collection activities in the areas with the highest densities, primarily the swampy lowlands [20], but hunters working in areas with low orangutan densities might have encountered very few or no orangutans and would rarely record such missing records, although during recent surveys such zero-encounters are commonly reported. Our separate analyses of encounters/non-encounters and conditional abundance (given at least one detection) allow us to reach some conclusions that are not affected by the possibility of changes in the pattern of underreporting non-encounters. The statistically significant outcomes of linear regression of non-zero encounter data, indicates that there is an overall decline in the numbers of detected orangutans, irrespective of possible biases in reporting non-encounters.

### Possible causes of declining orangutan encounter rates

A possible explanation for lower encounter rates in recent times is that because of increased frequency of encounters between people and orangutans, orangutans are now more elusive and have learned to avoid people. This would especially be the case if orangutans had learned to consider humans as a serious threat. Descriptions by Wallace [4] or Beccari [5] do suggest that orangutans were less likely to flee when they encountered humans than unhabituated orangutans encountered in the 21^st^ century. There is also some indication, although not substantiated by data, that in areas where orangutans have not been hunted for a long time, such as the Kinabatangan area in Malaysian Borneo, they tend to be easier to see than in areas where hunting still occurs. Still, because orangutans are not group living there is little to learn from other group members being shot. And also, because of their size and slow movement, orangutans have a high chance of being killed once spotted. Such characteristics would make it less likely that orangutans would learn to actively avoid people. A more detailed analysis of descriptions of both recent and historic orangutan encounters might reveal whether behavioural changes have indeed occurred. We doubt, however, that such changes could fully account for the observed decrease in encounter rates.

A more likely explanation for decreased encounter rates is that the local densities of orangutans have actually declined. This leads to the question of what would have caused this decline. We investigate the major causes of orangutan decline to assess how they relate to the observed decline in encounter rates: habitat loss and degradation, hunting, and disease.

**Figure 3:**
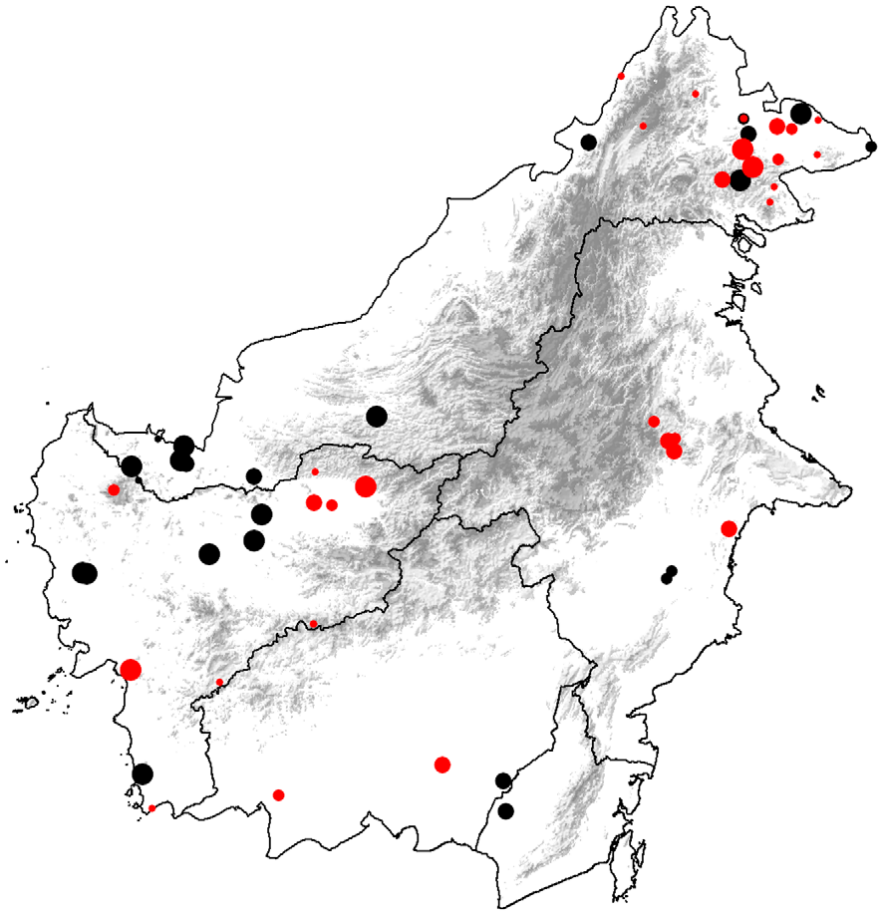
Map of Borneo with locations of surveys. The location of orangutan surveys conducted before (black symbols) and after (red symbols) large scale deforestation started (with cut-off year 1965), in relation to the upland areas of Borneo (indicated by the grey area in the centre of the island). Location symbols are scaled to daily encounter rates, with smallest symbol representing encounter rate = 0; next size, between 0 and 0.05; next size, between 0.05 and 0.22; and largest symbol, >0.22.

Although we avoided using disturbed sites in the analysis, it is possible that overall forest disturbance around sites surveyed in the 19^th^ century was lower than in the late 20^th^ and 21^st^. Deforestation has disproportionately affected lowland forests and orangutans disproportionately favor such habitats. If the 19^th^-century expeditions had focused on lowland forests, while modern fieldwork focused on Borneo's only remaining lightly-affected forests—those in the higher elevation interior—a decrease in encounter rates would also have been observed. Such a geographical shift is not obvious, however, with the focus in both pre- and post-deforestation surveys (with cut-off year 1965) being on coastal lowlands (Fig. 3). Also, if large-scale deforestation and forest degradation caused the observed differences in encounter rates, we would expect to see a sudden decline in encounter rates after the 1960s and 1970s, coincident with major intensification of these activities during this period. Our data suggest a decrease in orangutan encounter rates at least from the early 19^th^ century onward, some 120 years before major deforestation started [21], [22]. Unfortunately, we do not have enough data from the period 1900–1960/1970, to specifically test whether the decrease in encounter rates became more severe after large-scale deforestation started. What we do know, however, is that although orangutan numbers generally decrease following habitat disturbance [3], they do manage to survive in high densities in some areas that have been heavily disturbed or even clear-cut and planted with monocultural plantations [23]. We think therefore that the decline in encounter rates could not have been caused by reduced habitat quality alone, and that other factors need to be explored.

Hunting orangutans for meat or as agricultural pests remains common in most parts of Borneo, as shown from Borneo-wide surveys in the mid-1990s and 2008 [13], [24]. Because of teir low fecundity orangutans are very sensitive to hunting, and population viability models suggest that any population will go extinct eventually if hunting increases annual adult mortality by >1% [25]. Such local extinctions have been demonstrated by Pleistocene and Holocene orangutan remains found in caves in parts of Borneo where orangutans no longer occur, although suitable habitat remains [26], [27]. In fact, thousands of orangutan teeth found in sub-recent deposits in areas where orangutans had become extinct by historic times (Niah in north-western Borneo, and Padang in central Sumatra) suggest that orangutans were as commonly hunted as the ubiquitous wild pigs. Thus, in many areas, orangutans had already become extinct or reduced to very low population levels by the time of the first orangutan distribution assessments in the 19^th^ century. Spatial patterns show that local extinctions of orangutan populations that occurred before the time of the first descriptions of their ranges had primarily occurred in areas with nomadic human societies [13]. These people generally roam in upland areas with poor soils where permanent agriculture is difficult to maintain. The effective hunting ranges of these nomadic people were large, as opposed to settled agriculturalists that would have mostly hunted near their village and had less time to hunt. A combination of reduced carrying capacity in upland forests because of lower soil fertility, and higher hunting pressure may be an important explanation why orangutans became locally extinct in extensive dryland forest areas of Borneo during pre-historic times.

As opposed to dryland forests, freshwater and peat swamp environments of Borneo were virtually uninhabited by people until the 19^th^ century when commercial extraction of wood and forest products started [21]. Thus, we assume that these environments contained the majority of orangutans, not necessarily because these were ecologically more suitable, but rather because hunting in swamps had been rare or absent until the late 18^th^ and early 19^th^ century. These swamps are also the areas where most collecting during the 19^th^ century occurred (Fig. 3). We hypothesize that, once swamp areas started to be exploited commercially and human population densities increased, hunting of orangutans by local people as well as trophy collectors, and later for the pet trade, reduced densities in swamp habitats resulting in the observed reduced encounter rates.

The above hunting scenario is supported by genetic analyses of orangutan populations in the Malaysian state of Sabah, which strongly suggest that a decline in Sabah's orangutan populations of at least one order of magnitude most likely occurred within the last one or two centuries [28]. It is shown that the decline has been very recent and sharper than generally assumed. The current orangutan distribution in Sabah is also strongly related to the distribution of hunter-gatherers tribes according to interview data (MA unpubl. data). Orangutans are absent from most of the western side of the state where, until recently, they were heavily hunted for meat or traditional medicine, but they are common in the eastern forests of the state, that are either not inhabited or occupied by tribes with no primate hunting tradition. It remains unclear why hunting pressure would have increased in western Sabah a few hundred years ago. The timing coincides with the approximate end of head-hunting in Borneo, which had kept large parts of the island too dangerous to travel in [29], [30]. Banks [31] inferred that head-hunting provided wildlife a refuge, because large areas of forest were avoided by hunters fearful of roving bands of head-hunters. He noted that, immediately after the colonial ban on head-hunting was enforced, many forest areas became much safer to travel through, allowing hunters to travel further from villages, and leading to the rapid demise of, for example, the Sumatran rhinoceros *Dicerorhinus sumatrensis*. Also, it has been suggested that orangutan heads replaced human heads as trophies [32], which may also have added to hunting pressure on orangutans.

Hunting-related distribution patterns of orangutans have also been noted in the Indonesian part of Borneo. Te Wechel [33] reported that in the early 20^th^ century around the lower Barito River in southern Borneo, orangutans were rarely seen near villages, but remained common in the inaccessible swamps away from the rivers. In the same region, Lumholtz [29] found that in the lower reaches of the rivers almost no one lived and that orangutans were more common, as judged by their frequent calls and sightings. Further upriver, however, towards the centre of Borneo, people were more common and orangutans rare. More recent surveys have reported similar patterns: distance to the nearest village known to hunt orangutans is the strongest predictor of orangutan population density [34].

A final possible cause of density declines and encounter rates is disease. Orangutans suffer from a range of diseases, many of which also affect people [35]. Even though such disease can have high mortality rates on animals in captivity, there are no documented cases of disease epidemics in wild orangutan populations. With increasingly-frequent human-orangutan contact caused by shrinking habitat extent, we cannot exclude disease as a possible contributing factor to overall declining densities. Still, without any supporting evidence of disease as a major threat to wild orangutans we do not think it has played a major role in the observed encounter rate trends.

### Recommendations for management and research

This study would have benefited if both historic and recent encounter rate data had been available from the same localities. We cannot claim to have found all relevant survey and collection data and urge others to search for areas for which there are both good historic and recent reports on orangutan encounters. To determine the relationship between encounter rates and densities, it would be useful to design a survey covering areas of high, medium and low orangutan densities (as determined by formal line transect methods) in which reconnaissance walks are done specifically to find wild orangutans. Such data would allow an estimation of historic orangutan densities.

The findings in this paper could change our view of orangutans, because it is suggested that many Bornean populations could presently occur at densities well below those that would be imposed by food availability (i.e., densities are below the ecological carrying capacity). This suggests that some behavioural information on orangutans is biased by the fact that such populations were not studied at carrying capacity. We do not know enough about the ecological and behavioural flexibility of orangutans to predict how the species would react to significant density suppression, but we recommend that researchers keep this idea in mind when they study orangutans: How would the species behave if natural densities were 10 animals/km^2^? There is a need to interpret orangutan behaviour and ecology in the light of these new insights. To understand better how orangutan behaviour is affected by density, lessons could be learned from situations where orangutans exist in high densities, such as the fragmented populations of the Kinabatangan River in Malaysian Borneo. Comparative studies of breeding behaviour and social interactions in different density settings, and under different historic and present hunting regimes, may provide further insights into the ecology of orangutans under optimal ecological conditions. In turn, this would provide useful input into management of both *in-situ* and *ex-situ* populations.

### Conclusion

The usefulness of historic literature data for assessing population trends of threatened species is shown, not just by mapping historic ranges, but through estimation of encounter or catch rates. Even though statistical noise and bias are unavoidable in such assessments, the insights they provide may considerably change our views on the ecology of species and how to prevent their extinction. We hope that such studies are stimulated by this paper.

## Materials and Methods

We estimated the number of encounters for Bornean orangutans in three ways. First, we counted the number of orangutan encounters described by hunters or surveyors in detailed natural history accounts. Second, we counted the number of orangutan specimens collected by a particular hunter, as recorded in museum catalogues. This included the catalogue of primates in the Singapore Museum [36], the online mammal catalogues of the Smithsonian Institution, the Field Museum in Chicago, and the Museum of Comparative Zoology, Cambridge, and the catalogues of the Leiden Museum and Zoologische Staatssammlung München. Third, we sent a questionnaire to 30 researchers who have worked in orangutan habitats, asking them when, where and for how long they worked in a particular orangutan habitat; and how many different, wild, non-habituated orangutans they saw during their study period (with an assessment of the accuracy of their estimate).

We divided the number of orangutan encounters by the total number of days of a particular survey/hunting episode to obtain a daily encounter rate. For museum records, we estimated daily encounter rates by counting all specimens collected between the first and last date of a particular collection series. When on a particular day a specimen labelled ‘juvenile’ was collected but no female, we counted the juvenile record as 2 animals, because immature orangutans are invariably encountered together with a female [37]. This results in minimum estimates of daily encounter rates, as it only includes animals actually collected (and still available in museum collections). Additional orangutans seen, but not shot, are not included in this minimum estimate.

To assess the effect of survey effort, we recorded how many people were on each survey or hunting team. For the historic and museum records we searched the hunting accounts for clues about the size of the team. Collectors often kept field diaries or wrote books which revealed such information. For the recent records we asked the researchers directly with how many assistants they generally conducted their surveys.

Because it is suggested in some studies that orangutan densities decline in degraded forests [38], [39], we used records for which forest condition was only lightly affected by human disturbance. We determined this by selecting only those recent surveys that had been conducted in protected areas and forest reserves (note that protected areas did not yet exist in the 19^th^ century). Thus, we left out surveys from timber plantations, and areas that had been severely degraded by timber extraction or fire, even though high encounter rates had been reported from some of these areas. We realize that forests in some protected areas have also been degraded and that in few of these there is effective law enforcement [40], but we think this is an appropriate selection criterion for relatively undisturbed historic and present sites.

One important consideration when conducting meta-analyses of historical data is whether a particular data set should be included or excluded. While it is unlikely that we were able to completely avoid all sources of bias, we attempted to minimize their potential effects. Publication bias is one important factor. If hunters did not collect orangutans during a collecting expedition, they were unlikely to mention this in their records, and clearly no specimens would end up in museums. There are, however, a few historic accounts that specifically mention that no orangutans were seen, although the orangutan nesting platforms indicated that the species was locally present. The more recent surveys included quite a number of zero counts. Because we do not know how biased the reporting is regarding zero-counts, we analyzed the zero/nonzero counts (detection) separately from the nonzero counts (abundance) and report both results.

The first analysis involves fitting a logistic regression model relating the probability of detecting at least one orangutan during an expedition to year, expedition size and expedition duration. The second relates the logarithm of the daily abundance to time and expedition size.

To allow confidence to be assessed under alternative assumptions we quote full P values [41], [42] without Bonferroni correction procedures [43]. Analyses were done in - R (The R Foundation for Statistical Computing: http://www.R-project.org).

## Supporting Information

Figure S1 Encounter rates and expedition duration. The relationship between encounter/non-encounter and expedition duration (measured in log(Days)), showing the increasing probability of an encounter with increasing duration. The probability of an encounter is nearly one for expeditions of longer than 148.5 days (or 5 log(Days)).
(0.21 MB TIF)

Figure S2 Diagnostic plots for linear regression model of abundance. Diagnostic plots for the linear regression model relating log Abundance to year, duration (log(Days)) and size of the expedition (Person) conditional on at least one encounter during the expedition. The residual plot shows no curvature or outliers in the data but does show some evidence of increasing variability. This is arguably not as strong in the scale-location plot. The semivariogram shows no evidence of temporal dependence in the residuals and the QQ-plot is roughly linear.
(0.34 MB TIF)

Figure S3 Diagnostics for regression model with log(Density) as the response and log(Rate+1) as the covariate.
(0.35 MB TIF)

Table S1 Orangutan encounter data for Borneo
(0.23 MB DOC)

Table S2 Logistic regression model for probability of detecting at least one orangutan on an expedition, omitting Person and Year as variables
(0.04 MB DOC)

## References

[1] Pauly D (1995). Anecdotes and the shifting baseline syndrome of fisheries.. Trends Ecol Evol.

[2] Lotze HK, Worm B (2009). Historical baselines for large marine animals.. Trends Ecol Evol.

[3] Husson SJ, Wich SA, Marshall AJ, Dennis RA, Ancrenaz M, Wich SA, Atmoko SU, Setia TM, van Schaik CP (2009). Orangutan distribution, density, abundance and impacts of disturbance.. Orangutans: geographic variation in behavioral ecology and conservation.

[4] Wallace AR (1869). The Malay Archipelago.

[5] Beccari O (1904). Wanderings in the great forests of Borneo. From the English translation published by Archibald Constable & Co. Ltd, London, reprinted in 1986.

[6] Selenka E (1898). Menschenaffen. Studien über Entwickelung und Schädelbau. Erste Lieferung: Rassen, Schädel und Bezahnung des Orang utan.. Sitz Königl Preuss Akad Wissens Berlin.

[7] Johnson O (1966). Last adventure. The Martin Johnsons in Borneo.

[8] Davenport RK (1967). The orangutan in Sabah.. Oryx.

[9] Coomans de Ruiter L (1932). Uit Borneo's Wonderwereld. Schetsen over dieren en planten.

[10] Schaller GB (1961). The orangutan in Sarawak.. Zoologica.

[11] Hornaday WT (1885). Two years in the jungle. The experience of a hunter and naturalist in India, Ceylon, the Malay Peninsula, and Borneo.

[12] Hui C (2006). Carrying capacity, population equilibrium, and environment's maximal load.. Ecol Model.

[13] Rijksen HD, Meijaard E (1999). Our vanishing relative. The status of wild orang-utans at the close of the twentieth century.

[14] Singleton I, Wich SA, Husson S, Atmoko SU, Leighton M (2004). Orangutan Population and Habitat Viability Assessment: Final Report.

[15] Marshall AJ, Ancrenaz M, Brearley FQ, Fredriksson G, Ghaffar N, Wich SA, Utami SS, Mitra Setia T, van Schaik CP (2009). The effects of forest phenology and floristics on populations of Bornean and Sumatran orangutans: are Sumatran forests more productive than Bornean forests?. Orangutans: geographic variation in behavioral ecology and conservation.

[16] Wich SA, Buij R, van Schaik CP (2004). Determinants of orangutan density in the dryland forests of the Leuser Ecosystem.. Primates.

[17] Meijaard E, Nijman V, van Balen S (2005). The former status of the white-shouldered ibis *Pseudibis davisoni* on the Barito and Teweh rivers, Indonesian Borneo.. Raff Bull Zool.

[18] DeMatteo KE, Loiselle BA (2008). New data on the status and distribution of the bush dog (*Speothos venaticus*): Evaluating its quality of protection and directing research efforts.. Biol Cons.

[19] Hampel FR, Ronchetti EM, Rousseeuw PJ, Stahel WA (1986). Robust Statistics: The Approach based on Influence Functions.

[20] Mohnicke O (1883). Blicke auf das Pflanzen- und Thierleben in den Niederlandischen Malaianländern. III..

[21] Knapen H (2001). Forests of fortune? The environmental history of Southeast Borneo, 1600–1880.

[22] MacKinnon K, Hatta G, Halim H, Mangalik A (1996). The ecology of Kalimantan, Indonesian Borneo.

[23] Meijaard E, Albar G, Rayadin Y, Nardiyono, Ancrenaz M (in review). Unexpected ecological resilience in Bornean Orangutans and implications for pulp and paper plantation management.. PloSOne.

[24] Mengersen K, Meijaard E, Wells J, Christy L, Buchori D (In press). The sounds of silence: Listening to the villagers to learn about orangutans.. Significance.

[25] Marshall AJ, Lacy R, Ancrenaz M, Byers O, Husson S, Wich S, Atmoko SU, Mitra Setia T, van Schaik CP (2009). Orangutan population biology, life history, and conservation. Perspectives from population viability analysis models.. Orangutans: geographic variation in behavioral ecology and conservation.

[26] Harrison T (2000). Archaeological and ecological implications of the primate fauna from prehistoric sites in Borneo.. Indo-Pac Preh Ass Bull.

[27] Hooijer DA (1948). Prehistoric teeth of man and of the orang utan from Central Sumatra, with notes on the fossil orang utan from Java and Southern China.. Zool Meded Rijksmus Leiden.

[28] Goossens B, Chikhi L, Ancrenaz M, Lackman-Ancrenaz I, Andau P (2006). Genetic signature of anthropogenic population collapse in orang-utans - art. no. e25.. PLoS Biol.

[29] Lumholtz C (1920). Through Central Borneo. An Account of Two Years' Travel in the Land of Head-Hunters. Between the Years 1913 and 1917.

[30] Veth PJ (1854). Borneo's Wester-Afdeeling. Geographisch, Statistisch, Historisch; voorafgegaan door eene algemeene schets des ganschen eilands. 2 Vols.

[31] Banks E (1931). A popular account of the mammals of Borneo.. J Mal Br As Soc.

[32] Brooke J (1841). Letter relating to the orang-utan of Borneo.. Proc Zool Soc London.

[33] Wechel te G (1911). Iets over orang oetans.. Trop Nat.

[34] Marshall AJ, Nardiyono, Engstrom LM, Pamungkas B, Palapa J (2006). The blowgun is mightier than the chainsaw in determining population density of Bornean orangutans (*Pongo pygmaeus morio*) in the forests of East Kalimantan.. Biol Conserv.

[35] Russon AE, Wich S, Atmoko SU, Setia TM, van Schaik CP (2009). Orangutan rehabilitation and reintroduction.. Orangutans Geographic variation in behavioral ecology and conservation.

[36] Weitzel V, Yang CM, Groves CP (1988). A catalogue of primates in the Singapore Zoological Reference Collection. Department of Zoology, National University of Singapore.. Raff Bull Zool.

[37] Rijksen HD (1978). A fieldstudy on Sumatran orang utans (*Pongo pygmaeus abelii* Lesson 1827). Ecology, behaviour and conservation [PhD thesis].

[38] Felton AM, Engstrom LM, Felton A, Knott CD (2003). Orangutan population density, forest structure and fruit availability in hand-logged and unlogged peat swamp forests in West Kalimantan, Indonesia.. Biol Cons.

[39] Morrogh-Bernard H, Husson S, Page SE, Rieley JO (2003). Population status of the Bornean orang-utan (*Pongo pygmaeus*) in the Sebangau peat swamp forest, Central Kalimantan, Indonesia.. Biol Cons.

[40] Curran LM, Trigg SN, McDonald AK, Astiani D, Hardiono YM (2004). Lowland forest loss in protected areas of Indonesian Borneo.. Science.

[41] Day RW, Quinn GP (1989). Comparisons of treatments after an analysis of variance in ecology.. Ecol Monog.

[42] Stewart-Oaten A (1995). Rules and judgments in statistics: three examples.. Ecol.

[43] Nakagawa S (2004). A farewell to Bonferroni: the problems of low statistical power and publication bias.. Behav Ecol.

