# Supplementary figures and images for: Declining Orangutan Encounter Rates from Wallace to the Present Suggest the Species Was Once More Abundant
Source: PLoS One. 2010 Aug 11;5(8):e12042. doi: 10.1371/journal.pone.0012042 (PMC2920314; doi:10.1371/journal.pone.0012042)

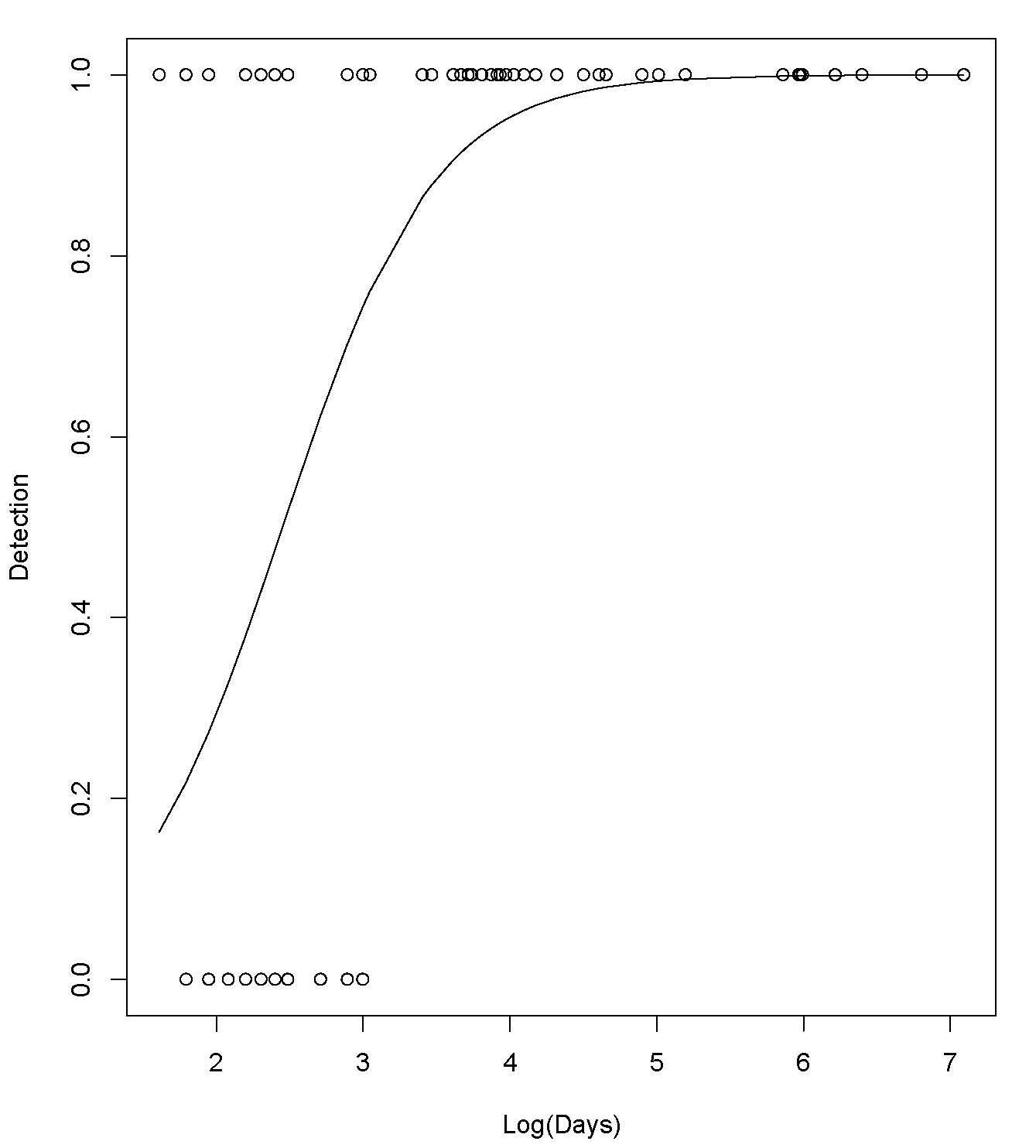

Supplement: Figure S1 — Encounter rates and expedition duration. The relationship between encounter/non-encounter and expedition duration (measured in log(Days)), showing the increasing probability of an encounter with increasing duration. The probability of an encounter is nearly one for expeditions of longer than 148.5 days (or 5 log(Days)). (0.21 MB TIF) [file pone.0012042.s001.tif]
