# Supplementary material for: Declining Orangutan Encounter Rates from Wallace to the Present Suggest the Species Was Once More Abundant
Source: PLoS One. 2010 Aug 11;5(8):e12042. doi: 10.1371/journal.pone.0012042 (PMC2920314; doi:10.1371/journal.pone.0012042)
Supplement: Table S1 — Orangutan encounter data for Borneo (0.23 MB DOC) [file pone.0012042.s004.doc]

**Supporting Information**

**Declining orangutan encounter rates** **from Wallace to the present suggest the species was once more abundant**

**Erik Meijaard, Alan Welsh, Marc Ancrenaz, Serge Wich, Vincent Nijman, Andrew J. Marshall**

**Table S1. Orangutan encounter data for Borneo**. T = total number seen and/or collected. M = mean number seen and/or collected per day. D = number of days spent in orangutan habitat. H = Hunting; S = survey. NP = National Park. ^1^ Selenka initially collected for 8 months in Borneo and Sumatra, but lost “part of his collection” due to shipwreck. During a second, 10 month collection trip “he almost compensated for the earlier loss”, resulting in a total number of “about 300” specimens from north-west Borneo. We do not know what part of the collection was lost and therefore how many specimens were collected in total—400 specimens is not unlikely. However, Büttikofer [1] reported that Moret, the Swiss hunter, with whom Selenka worked collected 139 specimens in 3 months on Mt. Kenepai alone; this is the number we use in the analysis.

^2^ Büttikofer [1] collected 7 specimens and described the encounter near the camp of several more orangutans. After making his initial collections he forbade his team to shoot more orangutans. The total number of orangutans encountered is therefore likely to be higher than the 10 used here.

^3^ These are likely to be underestimates of daily encounter rates, because they are based on the number of animals shot in the period between the first and last specimen, and thus not the total number of animals seen. Because hunters were primarily after adult animals, they would probably have seen more animals than they collected.

^4^ Abbott collected 26 orangutans skulls from the Sekaiam and Landak River areas in north-western Borneo (now stored in the collection of the Smithsonian National Museum of Natural History), but according to Hrdlička [2] only four of these had been killed by the collector’s hunters, whereas the rest had been obtained from a Dayak house.

^5^ These are accumulative numbers over the whole study period and it is unclear how many different individuals they represent. The contributors considered it likely that several of the subadult individuals they saw were actually the same. Hence, the value of 11 for Lubuk Kuali is most likely an over-estimate.

| **Period** | **Location** | **T** | **D** | **T/D** | **T/D/n** | **Collector or observer** | **H/S** | **Reported size of team (n = estimated team size)** | **Estimated local density** | **Source** |
| --- | --- | --- | --- | --- | --- | --- | --- | --- | --- | --- |
| 20 August 1836 – 1 December 1836 | South Borneo | 25 | 134 | 0.19 | 0.048 | S. Müller | H/S | 3 assistants (4) | NA | [3] |
| 1848 | Sadong River, Sarawak | 6 | 7 | 0.86 | 0.172 | James Brooke | H | With helpers but he shot all specimens himself (5) | NA | [4] |
| 1852-1854 | North-west Borneo | >50 | NA | NA | NA | O. Mohnicke | H/S | A few helpers and local hunters (10) | NA | [5] |
| 26 April – December, 1855 | South-west Sarawak | 29 | ~100 | 0.2 | 0.029 | A.R. Wallace | H | A few helpers and local hunters (7) | NA | [6,7] |
| 25 March – 1 May 1867 | South-west Sarawak | 26 | 37 | 0.7 | 0.100 | O. Beccari | H/S | 3-4 assistants and 2 hunters (7) | NA | [8] |
| 24 September – 1 October 1866 | Sarawak River | 0 | 8 | 0 | 0 | C. Collingwood | S | 13 people, including rowers | NA | [9] |
| 15 August - 1 December 1877 | Simujan River, Sarawak | 44 | 105 | 0.42 | 0.084 | Hornaday | H | A few assistants (5) | NA | [10] |
| 5 May 1889 – 1 June 1889 | Mt. Penrissen, Sarawak | 0 | 20 | 0 | 0 | RWC Shelford | H/S | About 10 in total (10) | NA | [11] |
| 1892-1896 | West Kalimantan north of Kapuas River | 139 ^1^ | 90 | 1.54 | 0.257 | M. Moret, collecting for E. Selenka | H | 1 or 2 professional collectors and local assistants (6) | NA | [1,12,13] |
| January 1893 | Sebangan and Sibuyan Rivers, Sarawak, Borneo | 40 | 30 | 1.33 | 0.333 | F.S. Bourns | H | 2 local assistants and a Malay guide (4) | NA | [14,15] |
| 2 January 1893 – 31 January 1894 | Mt. Kenepai, North-west Borneo | 10 ^2^ | 20 | 0.50 ^3^ | 0.125 | J. Büttikofer | H | 3 local collectors and team leader (4) | NA | [1,12], Leiden Museum Catalogue |
| 18 - 23 June 1905 | Sama River, North-west Borneo | 4 ^4^ | 6 | 0.67 ^4^ | 0.111 | W.L. Abbott | H | A few local hunters (6) | NA | [2,16], Smithsonian Mammal Collection Database |
| 18 March 1907 – 25 April 1907 | Landak River, North-west Borneo | 13 | ~37 | 0.35 ^3^ | NA | Bruegel | H | NA | NA | Zoologische Staatssammlung München |
| 2 -25 June 1907 | Kumpang River, nr Landak, North-west Borneo | 5 | 23 | 0.22 | NA | Bruegel | H | NA | NA | Zoologische Staatssammlung München |
| 18 June – 11 August, 1907 | South-west Borneo | 43 | 53 | 0.8 ^3^ | 0.080 | W.L. Abbott | H | A few helpers and local hunters (10) | NA | [17], Smithsonian Mammal Collection Database |
| 2 – 15 November 1907 | Landak River, North-west Borneo | 4 | 13 | 0.23 ^3^ | NA | Bruegel | H | NA | NA | Zoologische Staatssammlung München |
| 11 – 15 April 1909 | Ketungau River, North-west Borneo | 4 | 4 | 1 ^3^ | NA | Bruegel | H | NA | NA | Zoologische Staatssammlung München |
| October 1910 – June 1911 | Lower reaches of Barito and Kapuas Murung Rivers | 20 | 300 | 0.07 ^3^ | NA | P. te Wechel | H | NA | NA | [18]; Leiden Museum catalogue |
| 28 June – 29 July and 19 – 30 November 1925 | Kedang Kepala and Wahau Rivers | 1 | 41 | 0.02 | 0.003 | F.H. Endert | S | A few rowers and 2 guides (8 man) | NA | [19] |
| May – November 1925 | Kedang Kepala and Wahau Rivers | 1 | 42 | 0.02 | 0.004 | S. Siebers | S | 6 people | NA | [20] |
| 12 – 24 August 1929 | Sabah | 4 | 15 | 0.27 ^3^ | NA | F.C. Wonder | H | NA | NA | Field Museum collection |
| 16 June 1937 - 16 July 1937 | Sabah; Kinabatangan River, Camp Abai | 11 | 30 | 0.37 ^3^ | NA | S.L. Washburn and A. Schultz | H | NA |  | Museum of Comparative Zoology collection |
| 1 March-31 September 1954 | Sepilok and Lokan River, Sabah | 16 | ~200 | 0.08 | NA | R.K. Davenport | S | NA | NA | [21] |
| 3 weeks in 1959 | Various parts of northern Borneo | 3-5 | 21 | ~0.20 | 0.100 | K. Stott and C.J. Selsor | S | Just two people (2) | NA | [22] |
| 7 November 1960 – 2 January 1961 | Sarawak (near Batang Lupar River) | 10 | 56 | 0.18 | 0.18 | G.B. Schaller | S | No assistants (1) | NA | [23] |
| Early 1960s | Sarawak | 4 | 18 | 0.22 | 0.07 | B. Harrisson | S | 2 assistants (3) | NA | [24] |
| 1961-1963 | Dent Peninsula, Sandakan, and Kinabatangan | 5 | ~150 | 0.03 | 0.01 | N.S. Haile | S | 3 assistants (4) | NA | [25] |
| May 1963-July 1963 | Kinabatangan River area, | 1 | 48 | 0.02 | 0.005 | K. Yoshiba | S | Four people (4) | NA | [26] |
| 24 July 1963- 18 Aug. 1963 | Beaufort, Lahad Datu area, Klias | 1 | 9 | 0.11 | 0.037 | T. Okano | S | Three people (3) | NA | [27] |
| September 1967–November 1969 in. 27 individuals seen, max group size appears to be 3 | Segaliud-Lokan Forest Reserve, Sabah | 27 | ~500 | 0.05 | 0.008 | D.A. Horr | S | six experienced jungle men (6) | NA | [28] |
| 1968 | Kinabatangan, Sabah | 20 | ~150 | 0.13 | NA | D.A. Horr | S | NA | NA | [28] |
| June – October 1968; October 1969 – September 1970 | Ulu Segama, Sabah | 270 | 394 | 0.68 | 0.680 | J. MacKinnon | S | No assistants (1) | NA | [29,30] |
| November 1971 – November 1975 | Tanjung Puting | ~65 | ~1200 | 0.05 | 0.025 | B. Galdikas | S | small teams of 1-3 people (2) | 2.3 | [31] |
| July 1981 – January 1983 | Mentoko, Kutai, East Kalimantan | 20-22 | 390 | 0.05-0.06 | 0.050-0.060 | J. Mitani | S | 1 researcher with field assistants (1) | NA | [32,33] |
| May – June 1986 | Gunung Niut | 1 | 30 | 0.03 | NA | H. Simons | S | NA | NA | [34] |
| 14 months from April 1988 to July 1989 | Gunung Palung Nature Reserve | 20 | 350 | 0.06 | 0.015 | J. Mitani and D. Priatna | S | 2 researchers and 2 field assistants (4) | 4 | [35] |
| June 1989 – July 1990 | Gunung Palung NP | 9-10 | 350 | 0.03 | 0.015 | N. Makinuddin, pers. comm. | S | 2 | 3.5 | [36,37] |
| 1991 - 1992 | Gunung Palung NP | 15 | 400 | 0.04 | 0.020 | G. Paoli, pers. comm. | S | 2 | 3.5 | [36,37] |
| January 1994 | Kendawangan | 0 | 5 | 0 | 0 | Noor | S | NA | NA | [38] |
| 1994 – 1995 | Gunung Palung NP | 2 | 75 | 0.03 | 0.015 | G. Paoli, pers. comm. | S | 2 | 3.5 | [36,37] |
| 3 weeks between June and July 1995 | Lower Segama River | 1 | 21 | 0.05 | 0.013 | R. Rajaratnam | S | 3-5 people (4) |  | [39] |
| 1996 | East Betung Kerihun NP | 8 | 30 | 0.27 | NA | [40] | S | NA | 0.6 | [40,41] |
| March 1996 | Danau Sentarum | 1 | 5 | 0.2 | 0.05 | R. Dennis and R. Dennis, pers. comm. | S | team of 4 people (4) | 0.43 | [42] |
| 1997 | West Betung Kerihun NP | 0 | 50 | 0 | 0 | [40] | S | NA | 0.2 – 1.01 | [40,41] |
| 1999-2000 | Gunung Palung NP | 7 | 180 | 0.04 | 0.020 | B. Jarvis, pers. comm. | S | Alone or with 1 field assistant (2) | 3.5 | [36,37] |
| Sept 1996- August 1997, August 2000-August 2002. 2003. 2006, 2007, 2008 | Gunung Palung NP | 30-35 | 1100 | 0.03-0.04 | 0.020-0.027 | A. Marshall, in litt. | S | 1-2 (1.5) | 3.5 | [36,37] |
| July 1998 – June 2001 | Gunung Palung NP | ~35 | 600 | 0.06 | 0.03 | G. Paoli, pers. comm. | S | 2 | 3.5 | [36,37] |
| August 1999 – January 2000 | Gunung Palung NP | 16 | 45 | 0.36 | 0.240 | A. Felton and L. Engström | S | 1-2 (1.5) | 3.5 | [36,37] |
| June 2001 – October 2003 | S. Embaloh, Betung Kerihun NP | 2 | 37 | 0.05 | NA | [Takahashi in 41] | S | NA | 0.2 – 1.01 | [41] |
| December 2001 – February 2002 | Gunung Gajah, Berau District | 7 | 50 | 0.14 | 0.009 | L. Engström and team | S | 10-20 (15) | 3.27 | [43,44] |
| April – November 2002 | Sungai Gie, Berau District | 3 | 60 | 0.05 | 0.003 | L. Engström and team | S | 10-20 (15) | 0.71 | [43,44] |
| May 2001 | Gunung Rara FR (eastern part | 2 | 9 | 0.22 | 0.044 | M. Ancrenaz, in litt | S | 1 team of 5 people (5) | 1.5-3 | M. Ancrenaz, in litt |
| July 2002 | Ulu Kalumpang FR | 0 | 9 | 0 | 0 | M. Ancrenaz, in litt | S | 1 team of 5 people (5) | 0.4 | M. Ancrenaz, in litt |
| July 2002 | Madai Baturong FR | 0 | 9 | 0 | 0 | M. Ancrenaz, in litt | S | 1 team of 5 people (5) | 0.1-0.2 | M. Ancrenaz, in litt |
| September 2002 | Silabukan FR | 0 | 10 | 0 | 0 | M. Ancrenaz, in litt | S | 1 team of 5 people (5) | 0.5 | M. Ancrenaz, in litt |
| October 2002 | Trus Madi FR | 0 | 10 | 0 | 0 | M. Ancrenaz, in litt | S | 1 team of 5 people (5) | <0.5 | M. Ancrenaz, in litt |
| November 2002 | Poring FR | 0 | 11 | 0 | 0 | M. Ancrenaz, in litt | S | 1 team of 5 people (5) | 0.2 | M. Ancrenaz, in litt |
| January 2003 | Gomantong FR | 1 | 12 | 0.08 | 0.016 | M. Ancrenaz, in litt | S | 1 team of 5 people (5) | 3.1 | M. Ancrenaz, in litt |
| March 2003 | Ulu Tongud FR | 0 | 9 | 0 | 0 | M. Ancrenaz, in litt | S | 1 team of 5 people (5) | <0.3 | M. Ancrenaz, in litt |
| April 2003 | Segaliud-Lokan FR | 0 | 6 | 0 | 0 | M. Ancrenaz, in litt | S | 1 team of 5 people (5) | 1.1 | M. Ancrenaz, in litt |
| February 2005 | S. Sibau, Betung Kerihun NP | 0 | 9 | 0 | 0 | Ancrenaz and team | S | 6 people (6) | 0.27 | [41] |
| June-July 2005 | S. Embaloh, Betung Kerihun NP | 0 | 15 | 0 | 0 | Ancrenaz and team | S | 6 people (6) | 0.2 – 1.01 | [41] |
| November – December 1998 | Bukit Baka NP | 0 | 40 | 0 | 0 | A. J. Gorog, pers. comm. | S | NA | 0.1 | [45] |
| August 2003 – July 2005 | Sebangau | 33 | 600 | 0.06 | NA | H. Morrogh Bernard | S | NA | 2.5 | [46] |
| March – November 2006 | Several orangutan habitats in the Kelai watershed, East Kalimantan | 3 | 65 | 0.05 | 0.014 | C. Gordon and A.-M. Stewart, In litt., 20 November 2006 | S | 3-4 (3.5) | 1 | [43] |
| August 2006 – October 2006 | Sungai Lesan and Wehea, East Kalimantan | 3 | 32 | 0.09 | 0.045 | S. Spehar and P. Mathewson, in litt. 12 Jan. 2007 | S | Team of 2 (2) | 1 | [43,44] |
| May 2006 | Kulamba Wildlife Reserve | 0 | 7 | 0 | 0 | M. Ancrenaz, in litt | S | 2 teams of 5 people (10) | 2-3 | M. Ancrenaz, in litt |
| 2001 – 2006 | Kinabatangan | 35 | 750 | 0.05 | NA | M. Ancrenaz | S | NA | 2-4.4 | [47,48,49] |
| May – June 2003 | South Western Schwaner Range area | 0 | 18 | 0 | 0 | J. Ross & A. Hearn | S | 3 (3) | 0.46 | [45] |
| June – July 2003 | Bukit Baka Bukit Raya National Park | 0 | 12 | 0 | 0 | J. Ross & A. Hearn | S | 3 (3) | 0.1 | [45] |
| July – August 2003 | Sungai Tarantang | 0 | 15 | 0 | 0 | J. Ross & A. Hearn | S | 3 (3) | 0.1 | [45] |
| May 2005 | Danum Valley | 5 | 11 | 0.45 | 0.014 | M. Ancrenaz | S | 5-8 teams of 5 people (33) | 1 | M. Ancrenaz, unpubl. data |
| May 2005 | Malua-Segama FR | 2 | 11 | 0.05 | 0.010 | M. Ancrenaz, in litt | S | 1 team of 5 people (5) | 1-3 | M. Ancrenaz, in litt |
| Aug 2006 | Malua South | 0 | 10 | 0 | 0 | M. Ancrenaz, in litt | S | 2 teams of 5 people (10) | 0.7-1.4 | M. Ancrenaz, in litt |
| September 2006 | Malua North | 4 | 10 | 0.25 | 0.025 | M. Ancrenaz, in litt | S | 2 teams of 5 people (10) | 1.9-3.2 | M. Ancrenaz, in litt |
| 13 November 2006 – 6 April 2007 | Danum Valley | 8 | 51 | 0.16 | 0.080 | J. Ross & A. Hearn | S | 2 people (2) | 1 | M. Ancrenaz, unpubl. data |
| 30 April – 10 June 2008 | Different sites in East Kalimantan | 4 | 39 | 0.10 | 0.021 | G. Albar | S | 5 people (5) | NA | G. Albar, pers. comm. |

**References**

1. Büttikofer J (1896) Zoologische Skizzen aus der niederländischen Expedition nach Central-Borneo. Compt Rend Seanc Trois Cong Int Zool: 212-227.

2. Hrdlička A (1906) Anatomical observations on a collection of orang skulls from Western Borneo; with a bibliography. Proc US Nat Mus 31: 539-568.

3. Müller S (1857) Reis in het zuidelijk gedeelte van Borneo gedaan in het jaar 1836. In: Müller S, editor. Reizen en onderzoekingen in den Indischen Archipel gedaan op last der Nederlandsche Indische Regering tusschen de jaren 1828 en 1836 Vols 1 and 2. Amsterdam, The Netherlands: Koninklijk Instituut voor de Taal-, Land- en Volkenkunde van Nederlandsch-Indië. pp. 129-326.

4. Brooke J (1848) Narrative of events in Borneo and Celebes. Vol. 1. . London: J. Murray.

5. Mohnicke OGJ (1883) Blicke auf das Pflanzen- und Thierleben in den Niederlandischen Malaianländern. III. Das Thierleben. Münster: Natur und Offenbarung.

6. Wallace AR (1869) The Malay Archipelago. Oxford: Oxford University Press. 625 p.

7. Pearson MB (2005) A.R. Wallace's Malay Archipelago. Journals and Notebook. London: Linnean Society of London.

8. Beccari O (1904) Wanderings in the great forests of Borneo. From the English translation published by Archibald Constable & Co. Ltd, London, reprinted in 1986. Oxford, UK: Oxford University Press. 424 p.

9. Collingwood C (1868) Rambles of a naturalist on the shores and waters of the China Sea: Being observations in natural history during a voyage to China, Formosa, Borneo, Singapore, etc., made in Her Majesty's vessels in 1866 and 1867. London, UK: John Murray, Alblemarle Street.

10. Hornaday WT (1885) Two years in the jungle. The experience of a hunter and naturalist in India, Ceylon, the Malay Peninsula, and Borneo. London.

11. Shelford RWC (1916) A naturalist in Borneo. Kota Kinabalu, Malaysia: Natural History Publications (Borneo).

12. Jentink FA (1897) Note II. Zoological results of the Dutch scientific expedition to Central Borneo. The mammals. Notes Leyd Mus 19.

13. Selenka E (1898) Menschenaffen. Studien über Entwickelung und Schädelbau. Erste Lieferung: Rassen, Schädel und Bezahnung des Orang utan. Sitz Königl Preuss Akad Wissens Berlin 16: 1-92.

14. Bourns FS (1910) An orangutan hunt in Borneo. Bull Minn Acac Nat Sc 4: 173-181.

15. Timm RM, Birney EC (1980) Mammals collected by the Menage Scientific Expedition to the Philippine Islands and Borneo, 1890-1893. J Mammalogy 61: 566-571.

16. Lyon MW (1908) Mammals collected in western Borneo by Dr. W.L. Abbott. Proc US Nat Mus 33: 549-571.

17. Lyon MW (1911) Mammals collected by Dr. W.L. Abbott on Borneo and some of the small adjacent islands. Proc US Nat Mus 40: 53-146 + illustrations.

18. Wechel te G (1911) Iets over orang oetans. Trop Nat 1: 49-57.

19. Endert FH (1927) Reisverslag en floristisch verslag. In: Buys DW, editor. Midden-Oost Borneo Expeditie 1925. Weltevreden: G. Kolff & Co. pp. 135-312.

20. Siebers HC (1927) Midden-Oost Borneo Expeditie 1925 - zoologisch gedeelte. In: Buys DW, editor. Midden-Oost Borneo Expeditie 1925. Weltevreden: G. Kolff & Co. pp. 313-390.

21. Davenport RK (1967) The orangutan in Sabah. Oryx 20: 40-45.

22. Stott KJ, Selsor CJ (1961) The orang-utan in north Borneo. Oryx 6: 39-42.

23. Schaller GB (1961) The orangutan in Sarawak. Zoologica 46: 73-82.

24. Harrisson B (1987) Orang-utan. Oxford, UK: Oxford University Press.

25. Haile NS (1964) Orang-Human co-existence in North Borneo. Sarawak Mus J 11: 259-262.

26. Yoshiba K (1964) Report of the preliminary survey on the orang-utan in North Borneo. Primates 5: 11-26.

27. Okano T (1965) Preliminary survey of the Orang-utan in North Borneo. Primates 6: 123-128.

28. Horr DA (1972) The Bornean Orang Utan. Borneo Res Bull 4: 46-50.

29. MacKinnon J (1977) A comparative ecology of Asian apes. Primates 18: 747-772.

30. MacKinnon JR (1971) The orang-utan in Sabah today. Oryx 11: 141-191.

31. Galdikas B (1985) Subadult make orangutan sociality and reproductive behavior at Tanjung Puting. Amer J Primat 8: 87-99.

32. Mitani J (1985) Mating behaviour of male orangutans in the Kutai Reserve, Indonesia. Anim Beh 33: 392-402.

33. Mitani J (1985) Sexual selection and adult male orangutan long calls. Anim Beh 33: 272-283.

34. Simons H (1987) Gunung Niut Nature Reserve. Proposed Management Plan. Jakarta: WWF Indonesia. 42 + appendices p.

35. Mitani JC, Grether GF, Rodman PS, Priatna D (1991) Associations among wild orang-utans: sociality, passive aggregations or chance? Anim Beh 42: 33-46.

36. Felton AM, Engstrom LM, Felton A, Knott CD (2003) Orangutan population density, forest structure and fruit availability in hand-logged and unlogged peat swamp forests in West Kalimantan, Indonesia. Biol Cons 114: 91-101.

37. Johnson AE, Knott CD, Pamungkas B, Pasaribu M, Marshall AJ (2005) A survey of the orangutan (*Pongo pygmaeus wurmbii*) population in and around Gunung Palung National Park, West Kalimantan, Indonesia based on nest counts. Biol Cons 121: 495-507.

38. Noor YR, Hanafia EW (1995) A preliminary survey on the ecological potential of the Cagar Alam Muara Kendawangan, West Kalimantan. PHPA and AWB. Bogor, Indonesia p.

39. Rajaratnam R (1995) A mammal and bird survey in the lower Segama region, Sabah. Kota Kinabalu: Sabah Wildlife Department and WWF Malaysia.

40. Gurmaya KJ, Boeadi, Iskandar S, Susilo A, Sudradjat AR (1999) Keanekaragaman mamalia di Taman Nasional Bentuang Karimun, Kalimantan Barat. In: Soedjito H, editor. Rencana pengelolaan Taman Nasional Bentuang Karimun: Usaha Mengintegrasikan Kenanekaragaman Hayati dengan Pembangunan. Jakarta, Indonesia: WWF Indonesia, PHPA, ITTO. pp. 320-338.

41. Ancrenaz M (2006) Consultancy of survey design and data analysis at Betung Kerihun National Park, Indonesia. Jakarta: WWF Indonesia.

42. Russon AE, Meijaard E, Dennis RA (2000) Declining Orangutan populations in and around Danau Sentarum. Borneo Res Bull 31: 372-384.

43. Marshall AJ, Nardiyono, Engstrom LM, Pamungkas B, Palapa J, et al. (2006) The blowgun is mightier than the chainsaw in determining population density of Bornean orangutans (*Pongo pygmaeus morio*) in the forests of East Kalimantan. Biol Cons 129: 566-578.

44. Marshall AJ, Salas LA, Stephens S, Nardiyono, Engstrom L, et al. (2007) Use of limestone karst forests by Bornean orangutans (Pongo pygmaeus morio) in the Sangkulirang Peninsula, East Kalimantan, Indonesia. Am J Primatol 69: 212-219.

45. Singleton I, Wich SA, Husson S, Atmoko SU, Leighton M, et al. (2004) Orangutan Population and Habitat Viability Assessment: Final Report. Apple Valley, MN, USA: IUCN/SSC Conservation Breeding Specialist Group.

46. Morrogh-Bernard H, Husson S, Page SE, Rieley JO (2003) Population status of the Bornean orang-utan (*Pongo pygmaeus*) in the Sebangau peat swamp forest, Central Kalimantan, Indonesia. Biol Cons 110: 141-152.

47. Ancrenaz M, Calaque R, Lackman-Ancrenaz I (2004) Orangutan nesting behavior in disturbed forest of Sabah, Malaysia: Implications for nest census. Int J Primatol 25: 983-1000.

48. Ancrenaz M, Goossens B, Gimenez O, Sawang A, Lackman-Ancrenaz I (2004) Determination of ape distribution and population size using ground and aerial surveys: A case study with orang-utans in lower Kinabatangan, Sabah, Malaysia. Anim Cons 7: 375-385.

49. Ancrenaz M, Gimenez O, Ambu L, Ancrenaz K, Andau P, et al. (2005) Aerial surveys give new estimates for orangutans in Sabah, Malaysia. Plos Biol 3: e3.
