# Supplementary material for: Declining Orangutan Encounter Rates from Wallace to the Present Suggest the Species Was Once More Abundant
Source: PLoS One. 2010 Aug 11;5(8):e12042. doi: 10.1371/journal.pone.0012042 (PMC2920314; doi:10.1371/journal.pone.0012042)
Supplement: Table S2 — Logistic regression model for probability of detecting at least one orangutan on an expedition, omitting Person and Year as variables (0.04 MB DOC) [file pone.0012042.s005.doc]

**Supporting Information**

**Declining orangutan encounter rates** **from Wallace to the present suggest the species was once more abundant**

**Erik Meijaard, Alan Welsh, Marc Ancrenaz, Serge Wich, Vincent Nijman, Andrew J. Marshall**

**Table S2. Logistic regression model for probability of detecting at least one orang-utan on an expedition, omitting Person and Year as variables.** Logistic regression model for probability of detecting at least one orangutan on an expedition, omitting *Person* and *Year* as variables. Model 1c omits Person. Model 1d omits *Person* and *Year*. *Year* = year in which expedition was conducted. *Person* = number of people on an expedition. Log(*Days*) = natural logarithm of duration of expedition in days. Significance code: ‘**’: p < 0.01.

|  | Estimate | Std. Error | z value | Pr(>|z|) |
| --- | --- | --- | --- | --- |
| Intercept Model 1c | 20.709731 | 16.949276 | 1.222 | 0.22176 |
| Intercept Model 1d | -4.768 | 1.601 | -2.979 | 0.00289 ** |
| Year Model 1c | -0.012866 | 0.008592 | -1.497 | 0.13430 |
| log(Days) Model 1c | 1.928273 | 0.627266 | 3.074 | 0.00211 ** |
| log(Days) Model 1d | 1.945 | 0.601 | 3.237 | 0.00121 ** |
